# High Frequency Electromagnetic Radiation Stimulates Neuronal Growth and Hippocampal Synaptic Transmission

**DOI:** 10.3390/brainsci13040686

**Published:** 2023-04-19

**Authors:** Shaoqing Ma, Zhiwei Li, Shixiang Gong, Chengbiao Lu, Xiaoli Li, Yingwei Li

**Affiliations:** 1 School of Information Science and Engineering, Yanshan University, Qinhuangdao 066004, China; 2 Hebei Key Laboratory of Information Transmission and Signal Processing, Qinhuangdao 066004, China; 3 Institute of Electrical Engineering, Yanshan University, Qinhuangdao 066004, China; 4 Henan International Key Laboratory for Noninvasive Neuromodulation, Xinxiang Medical University, Xinxiang 453003, China; 5 State Key Laboratory of Cognitive Neuroscience and Learning, Beijing Normal University, Beijing 100875, China

**Keywords:** terahertz, neurons, dynamic growth, dendritic spine, synaptic transmission

## Abstract

Terahertz waves lie within the rotation and oscillation energy levels of biomolecules, and can directly couple with biomolecules to excite nonlinear resonance effects, thus causing conformational or configuration changes in biomolecules. Based on this mechanism, we investigated the effect pattern of 0.138 THz radiation on the dynamic growth of neurons and synaptic transmission efficiency, while explaining the phenomenon at a more microscopic level. We found that cumulative 0.138 THz radiation not only did not cause neuronal death, but that it promoted the dynamic growth of neuronal cytosol and protrusions. Additionally, there was a cumulative effect of terahertz radiation on the promotion of neuronal growth. Furthermore, in electrophysiological terms, 0.138 THz waves improved synaptic transmission efficiency in the hippocampal CA1 region, and this was a slow and continuous process. This is consistent with the morphological results. This phenomenon can continue for more than 10 min after terahertz radiation ends, and these phenomena were associated with an increase in dendritic spine density. In summary, our study shows that 0.138 THz waves can modulate dynamic neuronal growth and synaptic transmission. Therefore, 0.138 terahertz waves may become a novel neuromodulation technique for modulating neuron structure and function.

## 1. Introduction

Terahertz waves are electromagnetic waves that lie between the microwave and the far infrared, and their frequency is 0.1–10 terahertz (THz) [1,2,3]. Due to their low photon energy, light penetration, and fingerprint spectral properties, terahertz waves are used in a wide range of applications such as security detection, superconducting materials, and medicine [4,5,6,7]. In addition, terahertz waves are in the energy range for hydrogen bonding, charge transfer reactions, and van der Waals forces. This suggests that many of the rotational, oscillatory, torsional, and other energy levels of biological macromolecules (proteins, deoxyribonucleic acid (DNA), ribonucleic acid (RNA)) are only in the terahertz band [8,9,10,11]. Thus, terahertz waves of specific frequencies and energies can be coupled directly to proteins to induce coherent excitation to produce non-thermal effects [1,12].

Existing research shows that terahertz radiation interacts with hydrogen bonds in proteins [13], causing low-frequency molecular vibrations that lead to changes in the conformation and functional characteristics of the protein [8]. It can also cause non-thermal structural changes in protein crystals [14]. Additionally, it has been shown that terahertz radiation can precisely control the proton transfer process in the hydrogen bonding of base pairs, and can control DNA demethylation [15,16,17]. These studies suggest that terahertz waves can mediate changes in cell structure and function by exciting non-linear resonance effects in proteins and DNA. Based on this mechanism, terahertz waves of specific frequencies and energies affect neuron structure and function.

Currently, many scholars are beginning to focus on neurons’ responses to terahertz waves, but it is important to consider the safety of terahertz radiation protocols. Although terahertz waves are low in energy and do not ionize matter, this does not mean that they are safe [18,19]. Several studies have shown that terahertz waves’ effects on neurons are two-fold. For example, terahertz radiation (3.68 THz, 10–20 mW/cm^2^, 60 min) causes neuronal growth disorders [20]. When the terahertz radiation power was further increased (2.1 THz, 30 mW/cm^2^, 1 min), it resulted in a decrease in neuronal membrane potential with morphological disturbances and death after 2 h of radiation [21]. It has also been noted that terahertz radiation has no significant effect on either neuronal activity or survival [22,23]. These studies show that the effects of short-term terahertz radiation on the nervous system are nonlinear. However, studies on the safety of long-term and cumulative terahertz radiation on the nervous system are lacking.

Several studies have shown that terahertz radiation has positive effects on neuron structure and function. The growth of neuronal protrusions was promoted when neurons were radiated using broadband micro-terahertz (0.05–2 THz, 50 μW/cm^2^, 3 min). This promotion persisted when the power was reduced to 0.5 μW/cm^2^ [24]. M. I. Sulatsky et al. used terahertz waves (0.05–1.2 THz, 78 mW/cm^2^) to radiate chicken embryonic neurons for 3 min, and the results showed that terahertz radiation promoted neuronal protrusion growth [25]. However, the modulation mechanism of terahertz waves remains unclear. Further study has shown that terahertz radiation can promote neurite protrusion growth by altering the kinetics of gene expression associated with neurite growth [22,23]. However, neuronal growth and development is a dynamic, ongoing process and, to date, there are no studies which elucidate the long-term effects of terahertz radiation on dynamic neuronal growth.

Changes in neuronal structure usually lead to changes in neuronal function, and it has been shown that terahertz radiation can increase neuronal synaptic plasticity by promoting neuronal growth and regulating neurotransmitter release [23]. However, this research did not verify whether neuronal synaptic plasticity was altered. Other studies have entailed attempts to investigate the effects of terahertz radiation on neuronal function through electrophysiological experiments. At the microscopic level, terahertz radiation can increase intracellular Ca^2+^ and Na^+^ concentrations, and induce neuronal depolarization [26,27]. In addition, terahertz radiation can also reduce the neuronal membrane potential and affect the release rate of neuronal action potentials [20,28,29]. It has also been found that terahertz radiation alters neurons’ membrane resistance, which affects their excitability [22,30]. Neurons form the basis of a neural network and influence its properties [31]. At present, there is a lack of research on the effects and mechanisms of terahertz radiation on synaptic transmission and synaptic plasticity in neural networks.

In this study, we tried to ensure that sufficient terahertz was radiated to the samples while minimizing the effect of temperature variations on the experimental results. We measured the power of a 0.138 THz wave through an empty Petri dish containing 0.4–1 mL of culture fluid (at 0.1 mL intervals), and placed the dish in a mini incubator with controlled temperature, CO_2_ concentration and humidity. According to the pattern of neuronal growth and development, neurons were cultured in vitro for 2 days and then radiated several times using terahertz (20 min/day, 3 days), while recording neuronal growth and development on these days. In the study, neuronal cell body area and total protrusion length were used to characterize neuronal development and to analyze the effect of 0.138 THz radiation on dynamic neuronal growth and the cumulative effect. To investigate the safety of long-term neuronal radiation by 0.138 THz waves, we analyzed neuronal mortality after 3 days of terahertz radiation.

To further investigate the effect of 0.138 THz waves on the synaptic transmission efficiency of neural networks, we electrically stimulated the Sheffer lateral branch of isolated hippocampal slices to evoke a synaptic response in the CA1 region. At the same time, the postsynaptic potentials in the CA1 region were continuously recorded during terahertz radiation (60 min), and the slope and maximum amplitude of the postsynaptic potentials were used to characterize the efficiency of synaptic transmission in the CA1 region of the hippocampus. Finally, we analyzed the pattern of changes in the dendritic spine density of the cortical neurons in living rats after 0.138 THz radiation. This study demonstrates the 0.138 THz waves’ modulatory effect on cortical neuronal growth and the synaptic transmission efficiency in the CA1 region of the hippocampus. These results herald the potential development of 0.138 THz waves as a novel neuromodulation technique for intervention in neurodevelopmental disorders, and in Alzheimer’s disease.

## 2. Materials and Methods

### 2.1. Terahertz Irradiation Systems

**Figure 1:**
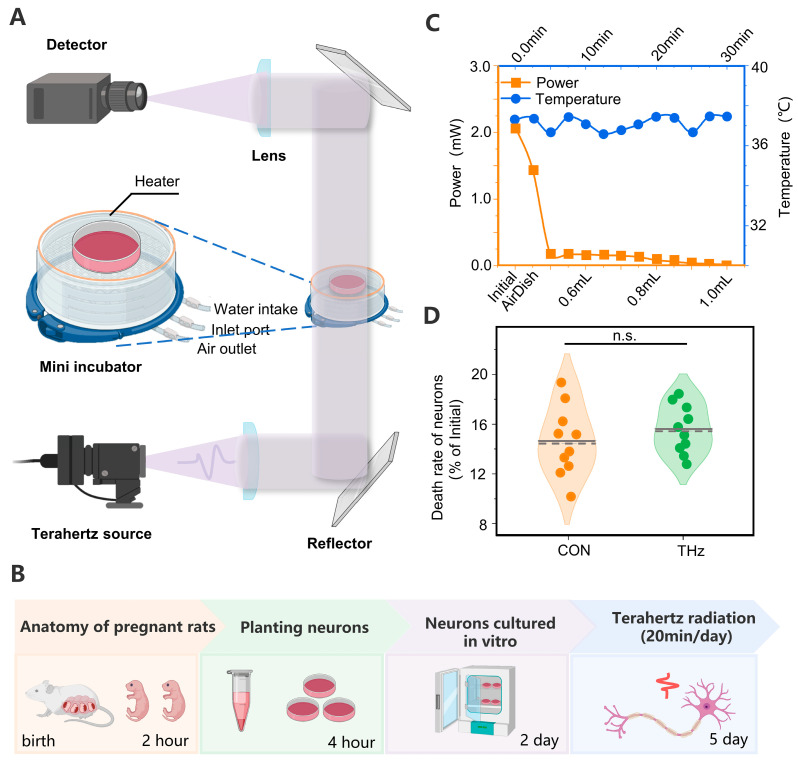
(**A**) Experimental platform for terahertz radiation cortical neurons. (**B**) Protocol for Terahertz Cumulative Radiation Neurons. The neurons were extracted and cultured in vitro for the first two days of the experiment. Waiting for the neurons to grow against the wall, the neurons were subjected to cumulative radiation for 3 days, 20 min/day. (**C**) Absorption characteristics and temperature variation patterns of the terahertz waves by neuronal culture fluid. (**D**) No significant difference in neuronal mortality in the Petri dishes after three days of terahertz cumulative radiation of the neurons. Data are mean ± SEM (*n* = 10 independent experiments). Independent samples *t*-test, n.s. indicates no significant difference.

The terahertz source used in this study was an avalanche diode terahertz source manufactured by TeraSense with an output frequency of 0.138 THz and a divergence of 8°. In order for the terahertz source to be compatible with multiple experimental platforms, the output optical path of the terahertz source was optimized. The terahertz radiation platform is shown in Figure 1A. We placed a Poly Tetra Fluoro Ethylene (PTFE) terahertz lens (LAT100, Thorlabs, Newton, NJ, USA) with a focal length of 100 mm at a distance of 100 mm from the terahertz source to convert the terahertz waves into parallel waves. A THz mirror (MAU50-6, Feichuang Yida, Beijing, China) with a thickness of gold coating sufficient to reflect incidental THz radiation was used to direct the beam orthogonally to the bottom surface of the culture plate. The effective area of the terahertz waves radiating into the Petri dish could be approximated as a circle with a diameter of 14 mm. The terahertz waves passing through the Petri dish were focused using a PTFE lens with a focal length of 100 mm, and the power of the transmitted waves was detected and recorded using a terahertz detector and power meter (RM9-THz, Ophir, Jerusalem, Israel). When radiating live rats with terahertz waves, we used two PTFE lenses to focus the terahertz waves, and the radiation area could be approximated as a circle with a diameter of 4 mm. Additionally, when isolating hippocampal slices with terahertz radiation, the terahertz waves’ effective radiation area could be approximated as a circle with a diameter of 14 mm.

### 2.2. Experimental Materials

Specific-pathogen-free Sprague Dawley (SPF SD) pregnant rats, at 12–15 days of gestation, were purchased from Beijing Vital River Laboratory Animal Technology Co., Ltd. (Beijing, China). The reagents required for cortical neuron culture and Golgi staining are shown in Table 1. The neurons were inoculated on 35 mm dishes pre-treated with 100 μg/L Poly-L-lysine and incubated at 37 °C, 5% CO_2_ incubator. After 4 h, the growing medium was replaced with a maintenance medium containing 97% neurobasal, 2% B27 and 1% glutamine. Two days later, the neurons were irradiated with terahertz for 20 min/day for 3 days.

### 2.3. Primary Neuron Cultures and Irradiation Protocol

Primary neuronal culture was based on Guo’s method [32,33], with slight modifications using SPF SD (specific-pathogen-free Sprague Dawley) pregnant rats, at 12–15 days of gestation, with bodyweight 300–350 g. The fetal rats’ cerebral cortexes were extracted in a sterile bench, cut up, added to Trypsin 0.25%, and then digested in an incubator for 15 min and removed every 3 min. Slowly and gently, we blew the neurons with a flame-passivated pasteurized dropper. The cell suspension was grown in 10% fetal bovine serum and 90% Dulbecco’s modified eagle medium, and adjusted to a concentration of 1 × 10^4^ cells in 1 mL. The neurons were then incubated in the incubator for 2 days, and after they had adapted to the environment and grown against the wall, they were irradiated for 20 min per day for 3 days. The experimental protocol is shown in Figure 1B.

### 2.4. Golgi Staining and Irradiation Protocols

To study the effect of terahertz radiation on neuronal dendritic spine density, we performed Golgi staining on the brain tissue [34,35]. We anaesthetized 50–60 g rats, placed them on a stationary table, and then radiated the brain tissue vertically from above using a terahertz system. The rats were sacrificed after 3 h of terahertz radiation, and the brain tissue was immediately removed and fixed in fixative for at least 48 h. The rat brain tissue was cut into 2–3 mm thick tissue blocks according to the part of the tissue to be observed. The brain tissue was gently rinsed three times with saline, placed in 45 mL round-bottomed PE tubes, submerged, and placed in a cool, ventilated location for 14 days, away from light (after 48 h of immersion, the new dye solution was changed, and this was repeated every 3 days for a total of 14 days). Next, we washed the tissue three times with distilled water and poured it into 80% glacial acetic acid to submerge it overnight. After that, we washed it with distilled water when the tissue was soft, and then placed it in 30% sucrose. In order to observe terahertz radiation’s effect on dendritic spine density in the rat motor cortex during the experiment, the rat brain tissue was coronally sectioned (Bregma 0.86 mm) with a section thickness of 100 um. The slices were attached to gelatin slides and left overnight to dry away from the light. After drying, the tissue slides were treated with concentrated ammonia for 15 min, washed with distilled water for 1 min, treated with acidic firm film fixative for 15 min, washed with distilled water for 3 min, dried, and then sealed with glycerin gelatine. Finally, we carried out imaging with a digital slide scanner (3D HISTECH, Panoramic 250, Budapest, Hungary).

### 2.5. Hippocampal Slice Preparation

Rats (SD) used in this experiment were sourced from the Institute of Medical Laboratory Animals, Chinese Academy of Medical Sciences, and bred in our animal facilities. The rats were kept in standard housing conditions: 4–5 rats per cage, with normal chow and water ad libitum, under a normal 6 am light/6 pm dark cycle [36]. The rats were anaesthetized with pentobarbital sodium and perfused with chilled (0 °C) sucrose-based cutting solution through the left ventricle until the limbs turned white. The brain was then rapidly removed and immersed in a chilled sucrose-based cutting solution containing (in mM): 225 sucrose; 3 KCl, 6 MgCl_2_, 1.25 NaH_2_PO_4_, 24 NaHCO_3_, 0.5 CaCl_2_, 10 glucose. Horizontal 350-lm-thick slices containing the ventral hippocampus were cut using a Leica VT1000S vibratome (Leica Microsystems UK, Milton Keynes, UK). Slices containing the ventral hippocampus were then transferred to an incubation chamber, where they remained submerged in oxygenated aCSF which consisted of (in mM) 126 NaCl, 3 KCl, 1.25 NaH_2_PO_4_, 2 MgSO_4_, 24 NaHCO_3_, 2 CaCl_2_, and 10 glucose, pH 7.35–7.45 at room temperature, until they were used for recording [37].

### 2.6. Electrophysiological Recordings and Irradiation Protocols

Brain tissue sections were placed on the incubation table, where they were perfused with aCSF at 32 °C, at a rate of 4 mL/min, with their surface exposed to warm, humidified carbogen (95% 2–5% CO_2_). Field potentials were recorded from SR of CA1, using a glass pipette filled with aCSF (resistance was 2–3 MΩ). The recordings were band-pass filtered online between 0.5 Hz and 2 kHz using an Axoprobe amplifier (Digitimer Ltd., Welwyn Garden City, UK) and a Neurolog system NL106 AC/DC amplifier (Digitimer Ltd., Welwyn Garden City, UK). The data were digitized at a sample rate of 10 kHz using a CED 1401 Plus ADC board (Digitimer Ltd.). Electrical interference from the mains supply was filtered from the extracellular recordings using HumBug noise eliminators (Digitimer Ltd.). Population Synaptic Potential (PSP) was evoked by orthodromic stimulation of the SCcommissural fibers in CA1 SR, using twisted 50 μm nickel/chromium wires. Pulses of 0.1 ms duration were delivered every 20 s. The standard stimulus intensity was set at the intensity that evoked a PSP amplitude of 30% of the maximum PSP amplitude. Data recording started when the induced PSP was stable, defining the first 10 min as the baseline, followed by terahertz radiation and simultaneous recording of the PSP signal for a duration of 60 min. After the end of terahertz radiation, data were recorded for 10 min.

### 2.7. Parameter Extraction and Analysis Methods

Firstly, to ensure that the neurons recorded were terahertz-radiated each time, and to find the same neuron quickly and efficiently, we drew two marker lines on the Petri dishes which intersected at the center of the dishes. Images were taken of neuronal growth and development in four quadrants, with the center of the Petri dishes as a right-angle coordinate system [38]. Next, we selected images with a clear background and low neuronal density (where the cell and protrusions of individual neurons were visible and not connected to other neurons). The images were opened using ImageView, the length of the neurite protrusion was measured using the arbitrary connection curve in the “Measure” menu, and the neurite cell face was measured using an arbitrary polygon. The area of the neuronal cell and the total length of the protrusion before terahertz radiation served as initial values. The neuronal cytosolic area and total protrusion length after 24 h of terahertz radiation were subtracted from the initial value and used as the growth value. By analogy, the increase in the neuronal cell area and the total protrusion length were calculated after days 2 and 3 of terahertz irradiation, respectively. Finally, the neuronal parameters in each dish were averaged to represent the cells in the whole dish, and were used as a sample value.

For the analysis of neuronal dendritic spine density changes, the Golgi-stained images were opened with CaseViewer. Since the attenuation of terahertz waves in brain tissue is large and the radiation area is limited, we only analyzed changes in the neuronal dendritic spine density in the M1 and M2 regions of the rat cortex at a depth of 200 um. We selected an area of 1000 μm^2^ and counted the neuronal dendritic spine density (ind/μm) in this area, then averaged it to represent the neuronal dendritic spine density in this area.

In analyzing the pattern of changes in synaptic transmission efficiency, we used the slope of descent and the maximum amplitude of the postsynaptic potential for characterization. The slope of the decrease in postsynaptic potential could be replaced by the slope of the line from 20% maximal to 80% maximal. The maximum amplitude represents the difference from the onset of postsynaptic potential generation to the nadir of the postsynaptic potential. The slope of descent and the maximum amplitude of the postsynaptic potential were extracted using a field potential signal analysis and processing system. 

### 2.8. Quantification and Statistical Analysis

The data in this study were first tested for normal distribution using the Shapiro–Wilk or Kolmogorov-Smirnov tests. All data are expressed as mean ± standard error of the mean (SEM), with specific cases noted separately. Significant differences between data sets were analyzed using independent sample *t*-tests or paired *t*-tests for data sets that conformed to the normal distribution. OriginPro performed the statistical analysis, and results were considered significantly different when the *p* < 0.05. The Supplementary Materials presents the raw data and statistical analysis results in Tables S1–S13.

## 3. Results

### 3.1. Time-Accumulated Irradiation with 0.138 THz Waves Does Not Cause Neuron Death

We found that the 0.138 THz wave was attenuated in the neuronal culture fluid. The penetration of the 0.138 THz waves was tested to ensure normal neuronal survival while minimizing the absorption of the 0.138 THz waves by the culture Petri dishes and the culture medium. The initial power was measured when the 0.138 THz waves did not pass the medium, and the power was measured after the 0.138 THz waves passed an empty Petri dish, and then 0.4–1 mL of culture medium. The measurement results are shown in Figure 1C. The results show that the initial power of the THz waves was around 2 mW, and the power after crossing the empty Petri dish was 1.8 mW. After adding 0.4 mL of culture fluid to the culture dish, the transmission power plummeted to 0.2 mW and decreased as the culture fluid volume increased, decreasing to 0.04 mW when the culture fluid volume was 1 mL.

The absorption of terahertz waves by the culture fluid caused the sample to warm. Thus, to exclude a series of reactions caused by changes in the temperature of the environment to which the neuron was exposed, we used a heater and a temperature sensor to create a feedback loop to stabilize the sample’s temperature at 37 °C. When the THz radiation was utilized to radiate the culture for 30 min, the temperature in the culture fluctuated around 37 °C. The measurement results are shown in Figure 1C.

Several studies have shown that THz irradiation can cause the disintegration of neuronal actin structures, structural damage to neurons, cell dehydration, and even death [20,39]. The causes of these phenomena are dependent on the terahertz waves’ frequency, energy, and radiation. To further verify the safety of the terahertz radiation protocol used in this study, we statistically analyzed the neurons’ mortality in the same area before and three days after terahertz radiation. The results showed that there was no significant difference in neuronal mortality between the control and terahertz groups after three days of irradiation. The statistical results are shown in Figure 1D.

### 3.2. Irradiation of 0.138 THz Promotes Dynamic Neuronal Cytosolic Growth

**Figure 2:**
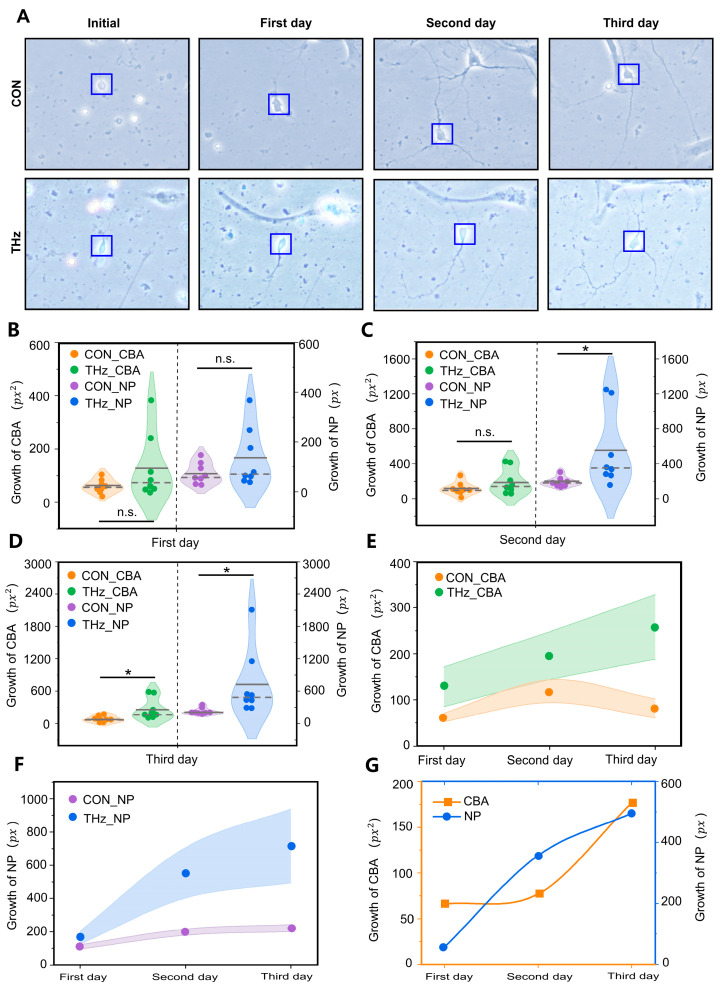
Terahertz radiation promotes the dynamic growth and development of cortical neurons. (**A**) Representative performance maps of neuronal developmental status in the terahertz (THz) and control (CON) groups. (**B**–**D**) On the second day of terahertz radiation, neurite protrusion (NP) growth was significantly higher than the CON. On the third day of THz radiation, the growth values of both NP and cell body area (CBA) were significantly higher than CON. Data are mean ± SEM (*n* = 8 independent experiments). *, *p* < 0.05 compared with control, n.s. indicates no significant difference. independent samples *t*-test. (**E**,**F**) The growth values of NP and CBA after THz radiation of the neurons were correlated with the number of days of radiation. However, this phenomenon was not observed in the CON. (**G**) Relationship between the radiation days in THZ_CBA minus CON_CBA and THZ_NP minus CON_NP.

To investigate the effect of 0.138 THz waves on the dynamic growth and development of neurons, we chose the neuronal cell area and total protrusion length as the statistical analysis quantities. To monitor whether the neurons were developing well during the experiment, the dynamic growth and development of the neurons were recorded photographically. The results showed that neurons in both the control and terahertz groups had already grown against the wall at the initial state, and had a small number of protrusions. The rapid growth of neuronal protrusions was observed after Day 2 in both the control and the terahertz groups, and the neurons remained well developed after three days of terahertz radiation, as shown in Figure 2A. The neuronal cytosol is the center of neuronal metabolism and nutrition, which is crucial to neuronal survival and development [40]. Therefore, we statistically analyzed the increase in neuronal cytosol area before and after terahertz radiation. The statistical analysis results are shown in Figure 2B–D. The growth value of the neuronal cell area was found to increase with increasing days of radiation in the terahertz group, but there was no similar phenomenon in the control group, as shown in Figure 2E. During the first two days of terahertz radiation, the mean neuronal cytosolic area was greater than that of the control group, but the statistical results were not significant, as shown in Figure 2B,C. On the third day of radiation, the growth value of the neuronal cytosolic area was significantly higher (258.1 ± 70.2 px^2^) than that in the control group (81.3 ± 20.5 px^2^), as shown in Figure 2D. To further analyze the neuronal cytosolic area growth values’ dependence on the terahertz radiation time, we calculated the difference in neuronal cytosolic area growth values between the terahertz group and the control group from the first day to the third day. We were surprised to find that this difference was correlated with the number of days of terahertz radiation, as shown in Figure 2G.

### 3.3. Radiation of 0.138 THz Promotes the Dynamic Growth of Neuronal Protrusions

Neuronal protrusions are the basis of information communication between neurons, and are the key to the formation of neural networks [41]. Therefore, we analyzed the growth values of the neuronal protrusions’ total length before and after statistically analyzing terahertz radiation. The statistical analysis results are shown in Figure 2B–D. A correlation was found between the increase in total neuronal protrusion length and the number of days in the control group, while the same phenomenon was observed in the terahertz group, as shown in Figure 2F. On the first day of terahertz radiation, the total neuronal protrusion length growth values in the terahertz group were not significantly different from those in the control group, as shown in Figure 2B. On both the second and third days of terahertz radiation, the total neuronal protrusion length growth was significantly higher in the terahertz group (554.2 ± 153.0 px, 716.1 ± 222.2 px) than in the control group (197.0 ± 18.5 px, 221.3 ± 20.9 px), as shown in Figure 2C,D. To investigate the total neuronal protrusion length growth values’ dependence on the terahertz radiation duration, we calculated the difference between the total neuronal protrusion length growth values of the terahertz group and the control group from the first day to the third day. The value of the total neuronal protrusion length growth in the terahertz group was correlated with the number of days of terahertz radiation, but there was no similar phenomenon in the control group, as shown in Figure 2F. Additionally, the difference in the growing value of the total neuronal protrusion length between the terahertz group and the control group was correlated with the number of days of terahertz radiation, as shown in Figure 2G.

### 3.4. Radiation of 0.138 THz Improves Synaptic Transmission Efficiency in the Hippocampal CA1

**Figure 3:**
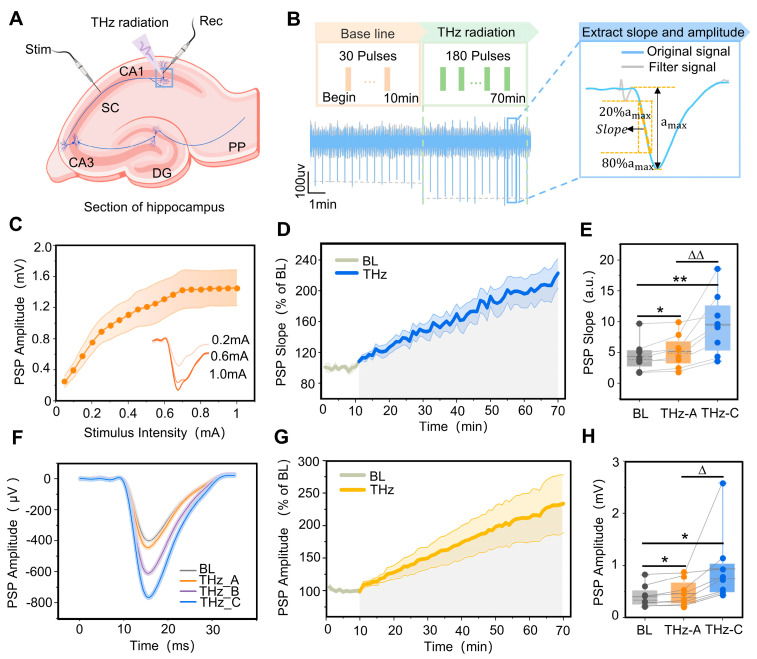
Terahertz wave modulation of synaptic transmission efficiency in the CA1 region of rat hippocampi. (**A**) Location of stimulation electrodes and recording electrodes in ventral sections of rat hippocampi. (**B**) Terahertz radiation protocol, and the method for extracting the slope and amplitude of postsynaptic potential (PSP) in the CA1 region of the hippocampus. (**C**) With the increase in stimulation intensity, the CA1 PSP amplitude gradually increased and stabilized near the maximum value. (**D**) The variation pattern of the CA1 PSP descent slope with terahertz radiation time, where the baseline (BL) is 10 min (grey curve) and terahertz radiation (THz) is 60 min (blue curve). The PSP descent slope was normalized. (**E**) The slopes of the PSP at 0–5 min (THz_A) and 55–60 min (THz_C) of terahertz radiation were significantly higher than the BL; THz_A < THz_C. Data are mean ± SEM (*n* = 8 independent experiments). *, *p* < 0.05. **, *p* < 0.01, compared with BL. ∆∆, *p* < 0.01, THz_C vs. THz_A, Paired *t*-test. (**F**) Representative maps of BL, THz_A, terahertz radiation 25–30 min (THz_B) and THz_C PSP in the hippocampal CA1 region. (**G**) The variation pattern of CA1 PSP amplitude with terahertz radiation time, where the BL is 10 min (grey curve) and THz 60 min (yellow curve). The PSP amplitude was normalized. (**H**) The amplitudes of the PSP at THz_A and THz_C were significantly higher than the BL; THz_A < THz_C. Data are mean ± SEM (*n* = 8 independent experiments). * *p* < 0.05, compared with BL, ∆, *p* < 0.05, THz_C vs. THz_A, paired *t*-test.

Synaptic transmission is an important physiological process that transmits information between neurons, and the kinetic properties of neuronal synaptic transmission are highly correlated with the structure of the neurons [42,43]. To further verify whether 0.138 THz radiation can also affect the synaptic transmission process in neurons, we used electrical stimulation of the Schaeffer collateral branch in isolated hippocampal slices to induce synaptic responses in the CA1 region, and analyzed the effect of terahertz radiation on the CA1 region’s synaptic transmission efficiency. The location of the stimulation electrode is shown in Figure 3A. The raw signal and PSP of the hippocampal CA1 region are shown in Figure 3B. To determine the standard stimulus intensity for each slice, stimulus–response curves were constructed using PSP amplitudes, as shown in Figure 3C. With increasing stimulus intensity, the PSP gradually stabilized near the maximum.

To quantify changes in SC-CA1 synaptic transmission efficiency, PSP’s slope and maximum amplitude were used for characterization, as shown in Figure 3B. To verify the SC-CA1 synaptic transmission efficiency’s dependence on the terahertz radiation time, the PSP slope’s variation pattern was quantified with the terahertz radiation time, as shown in Figure 3D. In the absence of terahertz radiation (the first 10 min), the PSP slope was stable around the baseline. The hippocampal CA1 region PSP slope continued to increase nearly linearly when terahertz radiation was applied, and this process lasted for 60 min. The slopes of the postsynaptic potentials at 0–5 min and 55–60 min of terahertz radiation (5.17 ± 0.95, 9.57 ± 1.78) were significantly higher than at the baseline (4.35 ± 0.87). Additionally, the PSP slope at 55–60 min was significantly higher than that at 0–5 min, as shown in Figure 3E,F. After terahertz radiation to the CA1 region of the rat hippocampus, the pattern of change in the maximum magnitude of PSP was similar to the change in slope, as shown in Figure 3G. The maximum magnitude of PSP at 0–5 min and 55–60 min of terahertz radiation (455.00 ± 87.04, 934.79 ± 250.03) was significantly higher than at the baseline (395.27 ± 76.03). Additionally, the maximum PSP magnitude was significantly higher at 55–60 min than at 0–5 min, as shown in Figure 3H.

**Figure 4:**
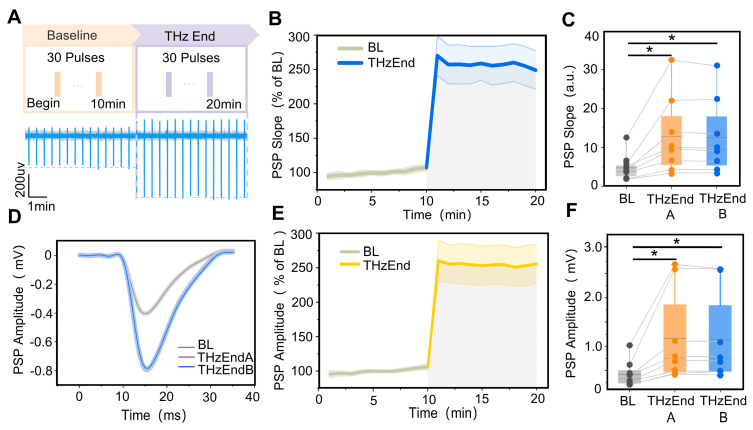
Terahertz radiation increases synaptic plasticity in rats’ hippocampal CA1 region. (**A**) Terahertz radiation protocol and raw signal. (**B**) The variation pattern of the CA1 PSP descent slope with terahertz radiation time, where the baseline (BL) is 10 min (grey curve) and the terahertz radiation end (THzEnd) is 10 min (blue curve). The PSP descent slope was normalized. (**C**) The slopes of the PSP at 0–5 min (THzEnd A) and 5–10 min (THzEnd B) were significantly higher than the BL. Data are mean ± SEM (*n* = 8 independent experiments) *, *p* < 0.05 compared with BL, paired *t*-test. (**D**) Representative performance maps of the BL, THzEnd A and THzEnd B PSP in the CA1 region of the hippocampus. (**E**) The variation pattern of CA1 PSP amplitude with terahertz radiation time, where the (BL) is 10 min (grey curve) and THzEnd is 10 min (yellow curve); the PSP amplitude was normalized. (**F**) The amplitudes of the PSP at THzEnd A and THzEnd B were significantly higher than the BL. Data are mean ± SEM (*n* = 8 independent experiments). *, *p* < 0.05 compared with BL, paired *t*-test.

To further investigate the duration of 0.138 THz radiation to improve the synaptic transmission efficiency in the CA1 region of the hippocampus, we analyzed the changes in the slope and amplitude of the postsynaptic potentials in the CA1 region within 10 min of the end of terahertz radiation. The terahertz radiation protocol is shown in Figure 4A. The results showed that the postsynaptic potential slope and amplitude were relatively stable within 10 min of the end of terahertz radiation, as shown in Figure 4B,D,E. Further statistical analysis revealed that the slope of postsynaptic potentials in the hippocampal CA1 region was significantly higher than the baseline (4.73 ± 1.19) at 0–5 min and 5–10 min (12.80 ± 3.51, 12.50 ± 3.39) from the end of terahertz radiation, as shown in Figure 4C,F. Additionally, the amplitude of postsynaptic potentials (1159.60 ± 334.47, 1142.70 ± 326.15) was significantly higher than the baseline (421.12 ± 97.04).

### 3.5. Radiation of 0.138 THz Increases Dendritic Spine Density in Cortical Neurons

**Figure 5:**
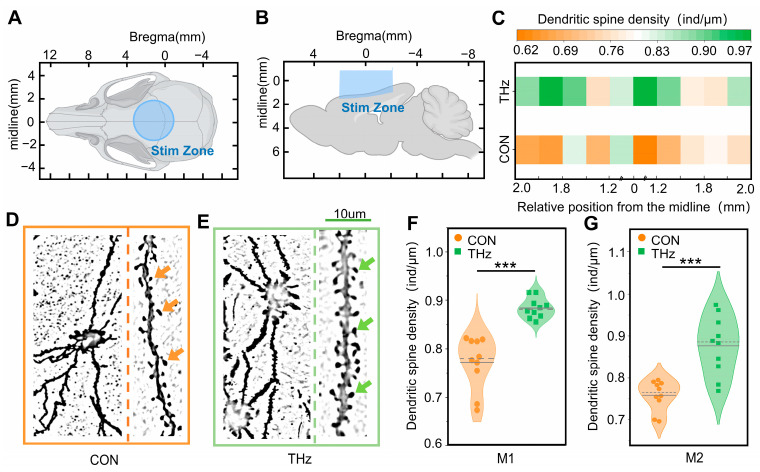
Terahertz radiation promotes the growth of neuronal dendritic spines. (**A**,**B**) Terahertz radiation to areas of the rat brains. (**C**) Patterns of dendritic spine density changes near the midline in coronal sections of rat brain tissue. (**D**,**E**) Golgi staining of neurons in the control (CON) and terahertz (THz) groups. (**F**,**G**) The cortical neuronal dendritic spine density was significantly higher in the rat cerebral cortexes (M1 and M2) than in the CON after 3 h of terahertz radiation. Data are mean ± SEM (*n* = 10 independent experiments); *** *p* < 0.001 compared with CON, independent samples *t*-test.

Neuronal dendritic spines are spiny protrusions on dendritic branches which are important sites for synapse formation in neurons. To an extent, changes in their density reflect changes in neuronal function [44]. To further investigate terahertz radiation’s effect on neuronal microstructure, we performed Golgi staining of the brain and analyzed the pattern of the terahertz radiation effect on the density of neuronal dendritic spines in the cerebral cortex. The terahertz radiation region can be approximated as a circle with a diameter of 4 mm; it is shown in Figure 5A,B. The results showed that the density of the dendritic spines near the midline of the coronal section of the rat brain tissue was higher than that of the control group after terahertz radiation, as shown in Figure 5C. The neurons after Golgi staining are shown in Figure 5D,E. Further statistical analysis showed that the cortical neuronal dendritic spine density in the rat cerebral cortex (M1 and M2) was significantly higher than that in the control group (0.77 ± 0.02, 0.76 ± 0.01) after 3 h of terahertz irradiation (0.88 ± 0.01, 00.87 ± 0.02), as shown in Figure 5F,G.

## 4. Discussion

Terahertz waves have the dual properties of photons and electrons with low electron energy and no ionizing effect on matter, but this does not mean that they are safe. In this study, we found that the time-accumulated radiation (20 min/day, 3 days) of neurons using 0.138 THz waves did not cause neuronal death, nor did it break the neurons’ dynamic growth pattern. From a physical point of view, terahertz radiation of neural biological tissues leads to biological effects essentially derived from the thermal and non-thermal effects of terahertz waves [1,45]. The thermal effect mainly comes from the strong absorption of terahertz waves by a large number of water molecules in the organism, while the non-thermal effect mainly comes from the nonlinear resonance effect of biological macromolecules excited by terahertz waves. Although individual terahertz photons do not have a direct ionizing damage effect on biological tissues similar to that of X-rays, the increase in the power of terahertz radiation leads to the warming of neurobiological tissues as it increases. This results in a range of biological effects [18,46]. For example, neurons exposed to terahertz radiation (3.1 w/cm^2^) for 30 min increased their cell temperature by over 7 °C, disintegrating the actin structure of the neuron and leading to neuronal growth disruption, dehydration effects, and damage to neuronal morphology [39]. Additionally, higher terahertz wave frequencies can cause biological tissues to warm. In contrast, we used a terahertz source with lower frequency and power (0.138 THz, 7 mW/cm^2^), reduced the culture fluid volume, and used a temperature feedback control loop to enable the neurons to survive in a 37 °C environment. We found that the effects of terahertz waves on neurons in this study were caused primarily by non-thermal effects.

The non-thermal effect of terahertz waves refers to the direct coupling of terahertz waves to biomolecules. This excites nonlinear resonance effects in biomolecules, causing conformational changes to them. The non-thermal effects of terahertz waves on biological tissues are also two-sided; for example, M.I. Sulatsky et al. used terahertz waves (0.05–1.2 THz, 928 mW/cm^2^) to radiate chicken embryonic neurons for 3 min, and inhibited the growth of neuronal protrusions [25]. However, when the power density was reduced to 78 mW/cm^2^, terahertz radiation promoted the growth of neuronal protrusions.

To date, no studies have demarked a safe threshold range for terahertz radiation to neurobiological tissues. However, higher terahertz radiation power and longer radiation duration can have some negative effects on neurons. The causes of this phenomenon may be thermal and non-effective, and an important parameter to distinguish between the two may be the total power of a single round of radiation. Therefore, in some studies, neuron safety has been ensured by reducing the terahertz radiation power. For example, there was no significant effect on neuronal survival after 15 min and 3 h of neuronal radiation using terahertz waves (3.1 THz, 70 μW/cm^2^) [22]. When the terahertz radiation power was low enough, radiation duration up to 3 h did not affect neuronal survival. However, the terahertz wave frequency might not be the main factor affecting neuronal safety. One study has shown that the use of 0.141 THz radiation on neurons for 1 h and 3 h significantly affected neither neuronal viability nor survival [23]. In this study, the total power of a single round of radiation was further reduced by using cumulative radiation over multiple days, and this did not significantly affect neuronal survival either. These studies show that the safety of terahertz radiation for neurobiological tissues is dependent on the terahertz wave energy and radiation duration, and there is a need to improve the safety evaluation index and further narrow the safety threshold range.

In this study, we found that cumulative terahertz radiation (20 min/day for 3 days) promotes the dynamic growth of cortical neuronal cell bodies. However, previous studies have shown that terahertz radiation of neurons for 20 min leads to a reduction in the neuronal cell area, and it has been suggested that this phenomenon is related to the cells’ dehydration effect [47]. In the present study, the change in neuronal cytosolic area was not due to the dehydration effect. In addition, there was a strong correlation between the effect of terahertz radiation on neuronal growth and the developmental cycle in which the neuron was located. However, the terahertz waves’ effect on neuronal growth and development was not significant when neurons were in a slow developmental state [38]. It has been shown that in vitro cultured neurons grow slowly at 6–24 h after inoculation, and during the latency period neurons undergo apposition and adaptation to their environment. After the latency period, the cells enter the rapid growth period, which usually occurs on the second to fifth day of cell inoculation [48]. Therefore, in this study, we chose to irradiate the neurons two days after inoculation, when the cells were in a rapid growth phase and the neuronal cytosolic area growth values after terahertz irradiation were significantly higher than those of the control group. To date, no studies have reported whether terahertz wave effects have a cumulative effect on neurons. In this study, we found that the difference between the neuronal cell area growth values of the terahertz group and the control group increased with the increase in terahertz radiation days. This indicated that terahertz radiation has a cumulative effect on neuronal cell growth promotion.

Recent studies have shown that terahertz radiation has a positive effect on neuronal protrusions, and one study showed that 0.14 THz radiation to hippocampal neurons for 30 min resulted in significantly longer neuronal protrusions in a terahertz group than in a control group [23]. In contrast, no significant effect on the growth of neuronal protrusions was found after Day 1 of terahertz radiation in our study. Although differences in radiation protocols can lead to differences in experimental results, the uniformity of data analysis and characterization parameters can facilitate comparability among studies. The characterization parameter in this study was the protrusion length, which may be subject to error if there is a difference in the initial lengths of the neuronal protrusions in the control and terahertz groups. The parameter in this study was neurite protrusion length, and this characterization may be subject to error if there is a difference in the initial lengths of the neurite protrusions between the control and terahertz groups. However, in this study, we used the growth value of the neuronal protrusions as the characterization parameter (current length–initial length), as this can mitigate the error caused by the difference in the initial neuron length.

Neuronal growth and development is a dynamic process, and a single round of radiation does not provide a complete characterization of the effects of terahertz radiation on dynamic neuronal growth and development. Therefore, in the present study, we statistically analyzed the dynamic growth of neuronal cytosol and protrusions during three days of terahertz radiation. We found no significant difference in neuronal protrusion growth values on Day 1 of terahertz radiation, compared to the control group. However, neuronal protrusion growth was significantly boosted on Days 2 and 3 of radiation. This phenomenon may be related to the developmental cycle of cultured neurons in vitro, where neuronal protrusions grow rapidly on Days 3–5 and were right within the faster growth phase on Days 2 and 3 of terahertz radiation [48]. The terahertz radiation did not alter the developmental cycle of the cultured neurons in vitro, and the facilitation effect on neuronal protrusions occurred only during the intrinsic growth cycle of neuronal protrusions. This study also revealed a cumulative effect of terahertz radiation on neuronal protrusion promotion. Similar phenomena have also been found, such as significantly higher synaptic protein expression on Day 6 compared to Day 4 of terahertz cumulative radiation [23].

No existing studies have clarified the mechanism by which terahertz waves regulate dynamic neuronal growth, but some studies have reported that terahertz radiation can alter gene expression associated with neuronal growth at the molecular level [23]. Other studies have reported that terahertz radiation can alter the proportional size of AMPA (α-amino-3-hydroxy-5-methylisoxazole-4-propionic acid) receptor isoforms at the protein level [38], thus affecting the permeability of AMPA receptor channels to Ca^2+^ [49]. Neuronal growth is correlated with intracellular Ca^2+^ concentration, where higher or lower concentrations prevent neuronal protrusions from growing while appropriate Ca^2+^ concentrations promote continuous protrusion growth [50]. Other studies have also directly demonstrated that terahertz radiation increases voltage-gated calcium channels’ permeability and increases intracellular Ca^2+^ levels in hippocampal neurons [26,51]. These findings suggest that terahertz waves may regulate the dynamic growth of neurons by altering neuronal receptor structures, gene expression and ion flow. However, there is no clear pathway to explain this phenomenon.

Previous experimental results have shown that terahertz radiation promotes the growth of neuronal protrusions, and this reflects an increase in the number of inputs individual neurons receive. This phenomenon is also reflected by an increase in dendritic spine density. Studies have shown a strong correlation between dendritic spines and neuronal synaptic transmission, synaptic plasticity, stress, learning and memory [52,53]. Moser et al. observed that spatial training increased the dendritic spine density of CA1 pyramidal neurons and improved learning ability in adult rats [52,54]. Wang et al. reported that radiofrequency electromagnetic radiation increased the dendritic spine density of neurons in the hippocampus and the prefrontal cortex of healthy mice, and improved recognition memory [4,53]. In this context, many studies have shown reduced dendritic spine density in hippocampal and cortical pyramidal neurons in Alzheimer’s disease models [55,56]. This implies that terahertz waves may regulate neuronal synaptic transmission and synaptic plasticity, and are a potential new strategy for anti-ageing and intervention in Alzheimer’s disease.

Consistent with the morphological changes, we found that 0.138 THz radiation increased the synaptic transmission efficiency in the CA1 region of the hippocampus. Meanwhile, some existing studies are consistent with our findings, for example, that at a more microscopic level, terahertz radiation can increase intracellular Ca^2+^ and Na^+^ concentrations, induce neuronal depolarization, and increase the frequency and amplitude of spontaneous synaptic transmission [22,26]. It is difficult to accurately characterize the changes in synaptic transmission efficiency with a single parameter. Therefore, in this study, we statistically analyzed the slope and amplitude of the decrease in postsynaptic potentials in the CA1 region of the hippocampus after terahertz radiation. We found that 0.138 THz radiation to the hippocampal CA1 region for 60 min, and PSP amplitude in the CA1 region, were correlated with the terahertz radiation time. This phenomenon may be related to receptor dynamics, where the magnitude of the synaptic response is directly dependent on the amount of receptor activation [57]. Thus, PSP amplitude can characterize changes in the neurotransmitter release amount, the number of receptors, and the number of neurotransmitter–receptor bindings [58,59,60]. This hypothesis is supported by several studies in which terahertz radiation decreased the inhibitory neurotransmitter GABA content in the neurons and increased the excitatory neurotransmitter Glu’s concentration [23]. In terms of changes in the number of receptors, it has been found that the number of AMPA receptors increased after terahertz radiation [38]. We also found that the slope of postsynaptic potentials in the CA1 region was correlated with the terahertz radiation time. The PSP slope has a strong correlation with AMPA and NMDA (*N*-methyl-D-aspartic acid) receptor kinetics, with AMPA receptor-mediated PSP alone peaking at 1–3 ms and recovering within 25 ms after stimulation [61]. In contrast, NMDA receptor-mediated PSP usually peaks around 10 ms and recovers 100 ms after stimulation [62,63,64]. The PSP we recorded was mediated by both AMPA and NMDA receptors, and therefore the slope was correlated with the proportion of these two processes involved. Thus, the increase in the PSP slope after terahertz radiation may be caused by an increase in the proportion of AMPA receptor-mediated PSP. Moreover, it has been found that the number of AMPA receptors increased significantly after terahertz radiation, but the number of NMDA receptors did not change significantly [38]. This finding corroborates our hypothesis about the slope of PSP after terahertz radiation. These studies suggest that 0.138 THz radiation improves the efficiency of synaptic transmission in the hippocampal CA1 region, which may be correlated with the neurotransmitter concentration, the number of receptors, and the ratio of AMPA and NMDA receptors mediating synaptic transmission. Moreover, 0.138 THz waves increase synaptic transmission efficiency in the hippocampal CA1 region as a slow and continuous process, and are highly correlated with radiation duration.

Current research on terahertz neurobiological effects remains in the exploratory stage, and therefore terahertz radiation safety is the first issue to be considered. Previous studies have shown that the effects of short-term terahertz radiation on the nervous system are nonlinear. In the present study, we explored long-term and cumulative terahertz radiation’s effects on neuronal survival. Since neuronal growth and development is a dynamic and continuous process, we investigated the effect of cumulative terahertz radiation on the dynamic growth and development of neurons, and analyzed the cumulative effect of terahertz radiation. Previous studies have shown that terahertz radiation can induce neuron depolarization and thus affect their excitability. On this basis, we investigated the patterns of terahertz radiation effects on hippocampal synaptic transmission and synaptic plasticity. To further investigate the mechanisms by which terahertz waves modulate the nervous system at a more microscopic level, we investigated terahertz radiation’s effects on neuronal dendritic spine density.

## 5. Conclusions

In this study, we investigated how safe 0.138 THz radiation is to neurons, and the long-term and cumulative effects of terahertz radiation on neuronal growth. We also explored the modulation pattern of 0.138 THz waves on hippocampal synaptic transmission and synaptic plasticity at the neural network level. The results showed that: (1) Cumulative radiation from 0.138 THz waves does not cause neuronal death, nor does it break their intrinsic growth pattern; (2) Cumulative radiation of neurons at 0.138 THz (20 min/day, 3 days) promotes the dynamic growth of neuronal cytosol and protrusions, and terahertz waves have a cumulative effect on neurons; (3) 0.138 THz waves improve hippocampal CA1 synaptic transmission efficiency, which is a continuous and slow process and highly correlated with terahertz radiation duration; (4) After terahertz radiation, the effect of increased synaptic transmission efficiency in the hippocampal CA1 area can last for more than 10 min; (5) At a more microscopic level, 0.138 THz radiation for 3 h in live rats also increased the neurons’ dendritic spine density. These results suggest that 0.138 THz can modulate dynamic neuronal growth and synaptic transmission. Therefore, 0.138 THz waves may become a novel neuromodulation technique for intervening in neurodevelopmental disorders and Alzheimer’s disease.

## Figures and Tables

**Table 1:** Main reagents required for cortical neuron culture.

Experimental Type	Reagent Name	Manufacturer	Item NO.
Neuron culture	Dulbecco’s Modified Eagle Medium	Gibco (New York, NY, USA)	11965092
	Neurobasal	Gibco	21103049
	B-27	Gibco	17504044
	Fetal Bovine Serum	Gibco	10099141C
	Trypsin 0.25%	Gibco	15050057
	Poly-L-lysine	Sigma (WA, USA)	P4832
	HBSS	Beynotime (Shangai, China)	C0218
	HEPES	Beynotime	ST090
Golgi staining	Fixative	Servicebio (Wuhan, China)	G1101
	OCT Embedding medium	Sakura (Torrance, CA, USA)	4583
	Golgi-cox staining solution kit	Servicebio	G1069
	Glycerin gelatin	Servicebio	G1402

## Data Availability

The raw data supporting the conclusions of this article will be made available by the authors, without undue reservation.

## Supplementary Materials

The following are available online at https://www.mdpi.com/article/10.3390/brainsci13040686/s1, Table S1: Growth of total length of neuronal protrusion and cell body area in the control group. Table S2: Growth of total length of neuronal protrusion and cell body area in the THz group. Table S3: Statistical description of te-rahertz radiation promoted neuronal growth. Table S4: T-test statistics of terahertz radiation pro-moted neuronal growth. Table S5: Slope and amplitude of postsynaptic potentials in hippocampal CA1 region before and after terahertz radiation. Table S6: Statistical description of slope and am-plitude of postsynaptic potentials in hippocampal CA1 region before and after terahertz radiation. Table S7: Paired T-test statistics of slope and amplitude of postsynaptic potentials in hippocampal CA1 region before and after terahertz radiation. Table S8: Slope and amplitude of postsynaptic potentials in the CA1 region of the hippocampus after cessation of terahertz radiation. Table S9: Statistical description of slope and amplitude of postsynaptic potentials in the CA1 region of the hippocampus after cessation of terahertz radiation. Table S10: Paired T-test statistics of slope and amplitude of postsynaptic potentials in the CA1 region of the hippocampus after cessation of te-rahertz radiation. Table S11: Dendritic spine density in the terahertz and control groups. Table S12: Statistical description of Dendritic spine density in the terahertz and control groups. Table S13: T-test statistics of Dendritic spine density in the terahertz and control groups.
