# Supplementary material for: High Frequency Electromagnetic Radiation Stimulates Neuronal Growth and Hippocampal Synaptic Transmission
Source: Brain Sci. 2023 Apr 19;13(4):686. doi: 10.3390/brainsci13040686 (PMC10136800; doi:10.3390/brainsci13040686)
Supplement: Supplementary file 1 [file brainsci-13-00686-s001.zip › brainsci-2261756-supplementary.pdf]

Table S1 Growth of total length of neuronal protrusion and cell body area in the control group.

Control	Growth of NP （px）			Growth of CBA （px^2）		
	**1 day**	**2 day**	**3 day**	**1 day**	**2 day**	**3 day**
**Sample 1**	96.65	235.26	201.60	105.50	258.75	4.50
**Sample 2**	132.39	160.09	347.83	27.12	79.75	160.50
**Sample 3**	174.67	185.46	202.71	53.81	103.75	62.62
**Sample 4**	70.01	162.65	270.25	53.50	95.75	162.83
**Sample 5**	150.88	194.93	173.61	87.00	169	84.75
**Sample 6**	89.99	185.12	179.98	38.00	100.83	99.00
**Sample 7**	89.52	307.79	202.82	59.00	116.50	53.00
**Sample 8**	67.75	145.04	191.26	69.75	23.25	23.50

* Neuronal Protrusion (NP), Cell Body Area (CBA).

Table S2 Growth of total length of neuronal protrusion and cell body area in the THz group.

THz	Growth of NP （px）			Growth of CBA （px^2）		
	**1 day**	**2 day**	**3 day**	**1 day**	**2 day**	**3 day**
**Sample 1**	101.75	1247.70	2119.90	241.00	425.00	562.00
**Sample 2**	382.59	1226.15	1137.86	381.83	417.00	584.90
**Sample 3**	269.11	517.31	519.57	65.00	131.41	234.45
**Sample 4**	107.97	296.69	288.27	51.06	56.26	148.43
**Sample 5**	205.06	359.37	537.43	82.75	102.75	148.50
**Sample 6**	80.29	280.59	279.60	113.00	212.00	108.75
**Sample 7**	78.47	343.32	409.23	42.00	63.50	109.33
**Sample 8**	99.00	162.31	436.73	47.75	157.00	168.75

* Neuronal Protrusion (NP), Cell Body Area (CBA).

Table S3 Statistical description of terahertz radiation promoted neuronal growth.

		Growth of CBA (px^2)				Growth of NP (px)			
		**ND**	**Mean**	**SEM**	**Median**	**ND**	**Mean**	**SEM**	**Median**
**1day**	**CON**	Yes	61.71	8.89	56.40	Yes	108.98	13.84	93.32
	**THz**	Yes	128.04	42.92	73.87	Yes	165.53	39.20	104.86
**2day**	**CON**	Yes	118.44	24.58	102.29	Yes	197.04	18.54	185.29
	**THz**	Yes	195.61	52.27	144.20	Yes	554.18	153.00	351.34
**3day**	**CON**	Yes	81.33	20.54	73.68	Yes	221,25	20.85	202.15
	**THz**	Yes	258.13	70.22	158.62	Yes	716.07	628.47	478.15

* Neuronal Protrusion (NP), Cell Body Area (CBA), Normal Distribution (ND), Standard Error of Mean (SEM).

Table S4 T-test statistics of terahertz radiation promoted neuronal growth.

	Growth of CBA (px^2)				Growth of NP (px)			
	**N**	**t**	**DF**	**P**	**N**	**t**	**DF**	**P**
**1day**	8	-1.51	14	0.15262	8	-1.36	14	0.19527
**2day**	8	-1.35	14	0.20289	8	-2.31	14	0.03615
**3day**	8	-2.41	14	0.02992	8	-2.21	14	0.04367

N=8 indicates that there are 8 pairs of data (THz=8, CON=8), P<0.05 indicates significant difference.

Table S5 Slope and amplitude of postsynaptic potentials in hippocampal CA1 region before and after terahertz radiation.

Sample	Slope（$a.u.$）			Amplitude（mV）		
	BL	THz-A	THz-C	BL	THz-A	THz-C
**Sample 1**	4.87	5.53	9.43	816.60	844.10	916.28
**Sample 2**	3.59	3.83	6.59	399.10	445.83	767.77
**Sample 3**	3.40	4.24	9.27	275.82	284.13	487.23
**Sample 4**	1.79	1.97	3.58	263.78	314.71	722.21
**Sample 5**	9.65	9.90	11.00	210.84	211.20	424.45
**Sample 6**	5.55	7.81	18.49	604.65	790.55	2579.73
**Sample 7**	4.08	5.63	14.09	387.57	514.40	1121.30
**Sample 8**	1.89	2.42	4.08	203.73	235.10	459.32

* BaseLine (BL), Terahertz radiation 0-5min (THz-A), Terahertz radiation 55-60min (THz-C).

Table S6 Statistical description of slope and amplitude of postsynaptic potentials in hippocampal CA1 region before and after terahertz radiation.

	Slope（$a.u.$）				Amplitude（mV）			
	ND	Mean	SEM	Median	ND	Mean	SEM	Median
**BL**	Yes	4.35	0.88	3.83	Yes	395.26	76.02	331.70
**THz-A**	Yes	5.17	0.94	4.89	Yes	455.00	87.04	380.27
**THz-C**	Yes	9.57	1.77	9.35	Yes	934.79	250.03	744.99

* Normal Distribution (ND), Standard Error of Mean (SEM).

Table S7 Paired T-test statistics of slope and amplitude of postsynaptic potentials in hippocampal CA1 region before and after terahertz radiation.

	Slope（$a.u.$）				Amplitude（mV）			
	N	t	DF	P	N	t	DF	P
**BL vs THz-A**	8	-3.12	7	0.01676	8	-2.63	7	0.03300
**BL vs THz-C**	8	-3.49	7	0.00999	8	-2.45	7	0.04382
**THz-A vs THz-C**	8	-3.56	7	0.00918	8	-2.49	7	0.04140

*N=8 indicates that there are 8 pairs of data (BL=8, THz-A=8, THz-C=8), $P<0.05$ indicates significant difference.

Table S8 Slope and amplitude of postsynaptic potentials in the CA1 region of the hippocampus after cessation of terahertz radiation.

**Sample**	**Slope（$a.u.$）**			**Amplitude（mV）**		
	**BL**	**THzEndA**	**THzEndB**	**BL**	**THzEndA**	**THzEndB**
**Sample 1**	5.16	9.96	9.80	405.56	792.71	783.72
**Sample 2**	3.59	6.62	6.43	275.82	492.53	481.35
**Sample 3**	3.40	9.51	9.21	263.78	715.33	700.84
**Sample 4**	12.36	32.43	30.94	1016.87	2685.77	2596.32
**Sample 5**	4.08	13.91	13.57	387.57	1109.38	1099.18
**Sample 6**	1.89	4.35	4.31	203.73	441.92	478.49
**Sample 7**	5.55	22.12	22.31	604.65	2610.78	2592.39
**Sample 8**	1.79	3.44	3.43	210.84	428.00	409.39

* BaseLine (BL), Terahertz radiation end 0-5min (THzEndA), Terahertz radiation end 5-10min (THzEndB).

Table S9 Statistical description of slope and amplitude of postsynaptic potentials in the CA1 region of the hippocampus after cessation of terahertz radiation.

	**Slope（$a.u.$）**				**Amplitude（mV）**			
	**ND**	**Mean**	**SEM**	**Median**	**ND**	**Mean**	**SEM**	**Median**
**BL**	Yes	4.73	1.18	3.83	Yes	421.10	97.03	331.70
**THzEndA**	Yes	12.79	3.50	9.74	Yes	1159.55	334.47	754.02
**THzEndB**	Yes	12.50	3.38	9.51	Yes	1142.71	326.14	742.28

* Normal Distribution (ND), Standard Error of Mean (SEM).

Table S10 Paired T-test statistics of slope and amplitude of postsynaptic potentials in the CA1 region of the hippocampus after cessation of terahertz radiation.

	**Slope（$a.u.$）**				**Amplitude（mV）**			
	**N**	**t**	**DF**	**P**	**N**	**t**	**DF**	**P**
**BL vs THzEndA**	8	-3.31	7	0.01290	8	-2.96	7	0.02095
**BL vs THzEndB**	8	-3.33	7	0.01256	8	-2.98	7	0.02044
**THzEndA vs THzEndB**	8	1.60	7	0.15277	8	1.38	7	0.20845

*N=8 indicates that there are 8 pairs of data (BL=8, THzEndA=8, THzEndB=8), P<0.05 indicates significant difference.

Table S11 Dendritic spine density in the terahertz and control groups.

Sample	Density of dendritic spines in M1 region（ind/um）		Density of dendritic spines in M2 region（ind/um）	
	**CON**	**THz**	**CON**	**THz**
**Sample 1**	0.673	0.855	0.696	0.768
**Sample 2**	0.686	0.862	0.700	0.784
**Sample 3**	0.755	0.867	0.746	0.827
**Sample 4**	0.771	0.875	0.755	0.845
**Sample 5**	0.776	0.878	0.756	0.883
**Sample 6**	0.783	0.885	0.773	0.888
**Sample 7**	0.815	0.889	0.783	0.899
**Sample 8**	0.816	0.894	0.787	0.932
**Sample 9**	0.819	0.916	0.790	0.962
**Sample 10**	0.822	0.916	0.795	0.974

* Control (CON), Terahertz radiation (THz).

Table S12 Statistical description of Dendritic spine density in the terahertz and control groups

	Density of dendritic spines in M1 region（ind/um）				Density of dendritic spines in M2 region（ind/um）			
	**ND**	**Mean**	**SEM**	**Median**	**ND**	**Mean**	**SEM**	**Median**
**CON**	Yes	0.77	0.01	0.78	Yes	0.75	0.01	0.76
**THz**	Yes	0.88	0.006	0.88	Yes	0.87	0.022	0.88

* Normal Distribution (ND), Standard Error of Mean (SEM).

Table S13 T-test statistics of Dendritic spine density in the terahertz and control groups

	Density of dendritic spines in M1 region（ind/um）				Density of dendritic spines in M2 region（ind/um）			
	**N**	**t**	**DF**	**P**	**N**	**t**	**DF**	**P**
**CON vs THz**	10	-6.13	18	8.5098E-6	10	-4.74	18	1.60671E-4

*N=10 indicates that there are 8 pairs of data (CON=10, THz=10), P<0.05 indicates significant difference.
